# Full length sequencing reveals novel transcripts of detoxification genes along with related alternative splicing events and lncRNAs in *Phyllotreta striolata*

**DOI:** 10.1371/journal.pone.0248749

**Published:** 2021-03-24

**Authors:** Guang Mao Shen, Shi Yuan Ou, Chu He, Jie Liu, Lin He

**Affiliations:** 1 Key Laboratory of Entomology and Pest Control Engineering, College of Plant Protection, Southwest University, Chongqing, China; 2 Academy of Agricultural Sciences, Southwest University, Chongqing, China; 3 State Cultivation Base of Crop Stress Biology for Southern Mountainous Land of Southwest University, Southwest University, Chongqing, China; Centro de Investigación y de Estudios Avanzados del I.P.N., MEXICO

## Abstract

The striped flea beetle, *Phyllotreta striolata* (Fabricius), damages crops in the *Brassicaceae*. The genetic data for this pest are insufficient to reveal its insecticide resistance mechanisms or to develop molecular markers for resistance monitoring. We used PacBio Iso-Seq technology to sequence the full-length transcriptome of *P*. *striolata*. After isoform sequence clustering and removal of redundant transcripts, a total of 41,293 transcripts were obtained, and 35,640 of these were annotated in the database of gene products. Structure analysis uncovered 4,307 alternative splicing events, and 3,836 sequences were recognized as lncRNAs. Transcripts with the complete coding region of important detoxification enzymes were further classified. There were 57 transcripts of P450s distributed in CYP2, CYP3, CYP4, and Mito CYP clades, 29 transcripts of ESTs from 4 functional groups, 17 transcripts of GSTs classified into 5 families, 51 transcripts of ABCs distributed in 6 families, and 19 transcripts of UGTs. Twenty-five lncRNAs were predicted to be regulators of these detoxification genes. Full-length transcriptome sequencing is an efficient method for molecular study of *P*. *striolata* and it is also useful for gene function analysis.

## 1. Introduction

The striped flea beetle, *Phyllotreta striolata* (Fabricius), is a pest damages economically important crops in the *Brassicaceae*. *P*. *striolata* adults feed on fresh leaves. They lay eggs in the soil, and the larvae feed on plant roots until pupation. After eclosion, the adults emerge from the soil and begin a new generation. *P*. *striolata* is widely distributed in the northern and western parts of the USA and is a major pest on cruciferous vegetables and oil producing *Brassica* cultivars [1,2]. Outbreaks of *P*. *striolata* now occur in southern China. The large populations of *P*. *striolata* have caused serious economic losses there. The small body size, strong mobility, and high reproductive rate of *P*. *striolata* have made it difficult to control [3]. Chemical pesticides are mainly used for controlling *P*. *striolata* but some populations have developed reduced sensitivity to common insecticides such as the neonicotinoids. Evaluation of new pesticides against this pest to determine efficacy has been expensive [4]. Little is known about resistance mechanisms of *P*. *striolata*, which can lead to reduced pesticide effectiveness and increased chemical use. It would be useful to obtain genetic information on the important insecticide targets and detoxification enzymes of *P*. *striolata*.

Few genetic data of *P*. *striolata* are available and less than 500 nucleotide or protein sequences have been submitted to the NCBI database. These data are insufficient to analyze the physiological characteristics of *P*. *striolata*. Whole genome analysis is a good way to obtain bioinformatics, but it not suitable for every species due to its high cost and long experimental period (including sample preparation, sequencing, and data analysis). As an alternative method, transcriptome analysis is often used in biological research of animals, plants, microorganisms, and insects. Next generation sequencing (NGS) technologies, such as ABI SOLid, Illumina Solexa, and Roch 454 systems, are commonly used in transcriptome sequencing [5]. The initial transcriptome analysis of *P*. *striolata* was performed by the Illumina platform, and this mainly focused on the chemoreception genes related to olfactory recognition [6]. With the advantages of high accuracy, throughput and sensitivity, and relatively low cost, Illumina sequencing has become a popular choice for transcriptome analysis. However, one disadvantage of Illumina sequencing is the short read length, which presents an obstacle for the study of specific gene functions [7]. Most genes obtained from Illumina sequencing are only partial sequences. Thus, RACE technology should also be used to obtain the full sequence of important genes. To overcome these limits, third generation sequencing (TGS) technology was invented based on single-molecule real-time (SMRT) sequencing technology [8]. With significantly longer reads (maximum of >20 000 nt) than NGS, full length transcripts can be easily screened out of the sequence data obtained from the PacBio platform. The complete transcripts also provide information useful for investigation of alternative splicing, alternative polyadenylation, fusion transcripts, long non-coding RNAs (lncRNA), and novel genes. With its moderate cost, TGS has become a good choice for researchers to obtain complete genetic information of specific species lacking whole genome data [9–11].

In this study, PacBio Iso-Seq technology (PacBio, Menlo Park, CA, USA) was used to sequence the full-length transcriptome of *P*. *striolata*. We classified transcripts with the complete coding region of important detoxification enzymes, analyzed alternative splicing events, and predicted lncRNA as a regulator of these transcripts.

## 2. Materials and methods

### 2.1. Beetle collection and RNA isolation

*P*. *striolata* were collected from the field in BiShan, ChongQing, China. Original host was *Brassica juncea*, and adults were reared on Chinese cabbage (*Brassica rapa L*. *ssp*. *pekinensis*). Total RNA was extracted from 20 adults (10 males and 10 females) using Trizol reagent (Invitrogen, CA, USA), and mixed together. The concentration of total RNA was quantified by Eon microplate spectrophotometer (BioTek, VT, USA), and OD_260/280_ and OD_260/230_ were test to assess purity. The integrity of RNA was confirmed by 1% agarose gel electrophoresis. Then, RNA sample was sent to Biomarker Technologies (Biomarker, Beijing, China) for full transcriptome sequencing on Pacbio platform. Since no dangerous species or technology were involved in this research, no permits were required for the work.

### 2.2. Library construction and full-length transcriptome sequencing

Ten μg total RNA with high quality (purity, concentration, and integrity) was used to construct the cDNA library. Full length cDNA was synthesized from mRNA by using SMARter^™^ PCR cDNA synthesis Kit (Clontech, CA, USA). Briefly, mRNA molecules were collected by oligo(dT) magnetic beads, and then were copied into first and second cDNA by using random hexamers, dNTP, RNase H, and DNA polymerase. Synthesized cDNA was purified by AMPure XP beads (Beckman Coulter, CA, USA), and modified with end repair, A tail and adapter ligation, exonuclease digestion. After quality assessment, the cDNA library was sequenced on the Pacbio platform.

### 2.3. Sequence assembly and assessment

Sequencing by the PacBio platform can provide information of an entire single transcript with extreme long reads (median 10 kb), which could be directly used to assemble a full-length transcriptome [12]. All of the original sequences were converted into Circular Consensus (CCS) sequences according to the conditions that full passes ≥1 and sequence accuracy >0.9. CCS sequences with 5’ primer, 3’ primer and polyA tail (if any) were predicted to be full length sequences, or else non full sequences.

Same transcripts may generate similar sequences, and these sequences were classified into one cluster. Each cluster had only one consensus sequence. Then, Cd-hit was used to remove redundancy to obtain non-redundant sequences [13]. Sequences with consistent exons except the 5’ end exon were merged to obtain the longest sequences as final transcripts [14].

### 2.4. Prediction of coding sequences and function annotation

The Open Reading Frame (ORF) was predicted by TransDecoder based on sequence length, log-likelihood score, amino acid sequence, and protein domain sequence in the Pfam database [15]. Transcripts containing a complete ORF were compared in main protein databases such as NR and COG by BLAST software to get annotation information [16].

### 2.5. Alternative splicing (AS) analysis

AS events in non-redundant sequences were predicted by Astalavista software with default parameters [17]. Sequences meeting the following conditions were considered as AS events: a) two sequences were both >1000 bp, and contained two high-scoring segment pairs. b) The alternative splicing gap was >100 bp, and splicing site location in relation to the 3’/5’ end was > 100 bp. c) Overlap of 5 bp was acceptable. Five different major types of AS events were classified as Intron retention, Exon skipping, Alternative 3’ splice site, Mutually exclusive exon, and Alternative 5’ splice site [18].

### 2.6. lncRNA and its target prediction

The coding potential of transcripts was screened to determine whether they were lncRNA or not. Four lncRNA analysis tools were comprehensively used to get a precise result. Transcripts recognized as lncRNA were confirmed simultaneously by the Coding-Non-Coding-Index (CNCI) [19], Coding Potential Calculator (CPC) [20], Coding Potential Assessment Tool (CPAT) [21], and Pfam [22]. The lncRNA–mRNA interactions were identified by LncTar, which was a reliable tool to efficiently predict the RNA targets of lncRNAs on a large scale [23].

### 2.7. Identification and analysis of detoxification related genes

Detoxification related genes (P450, EST, GST, ABC transporter, UGT) were screened out from the annotated sequences against the protein database. The complete coding region was further confirmed by the ORF finder (http://www.ncbi.nlm.nih.gov/gorf/gorf.html) and protein BLAST results. Genes from other insects such as *Tribolium castaneum* were used as reference to construct phylogenetic trees with MEGA software (neighbor-joining method and bootstrap analysis with 1000 replications) [24].

### 2.8. Sequence validation of transcriptome data by PCR

Primers were designed based on transcriptome data to amplify CDS of detoxification genes (S1 Table). The T3 Super PCR Mix Kit (TsingKe biological technology, BeiJing, China) was used to perform PCR. The PCR products were purified from 1% agarose gel by PCR Clean-Up System (Promega, Madison, WI, USA), and cloned into a pGEM-T Easy vector (Promega, Madison, WI, USA), then, recombinant plasmid were sequenced for nucleotide information (BGI, Beijing, China).

## 3. Results

### 3.1. Sequencing assembly

The clean data size of full-length transcriptome sequencing of *P*. *striolata* based on PacBio SMRT (single molecule real time) sequencing technology was 45.29 Gb. According to the condition that full passes ≥1 and sequence accuracy >0.9, 475,276 circular consensus (CCS) sequences were extracted from the clean data. The total reads of CCS and mean read length were 1,307,818,400 and 2,751. 400,368 CCS sequences (84.24% of total) containing a correct 5’ primer, 3’ primer, and polyA tail, and were classified as full-length non-chimeric sequences. Same full-length non-chimeric sequences were screened out and divided into a cluster. Only one consensus isoform was reserved in each cluster. In this step, 76,994 consensus isoforms were detected with an average read length of 2,356 bp. Although 97.65% of these isoforms (75,182) had high quality (accuracy >0.99), multiple copies of same transcripts were still likely to be placed in different clusters, which resulted in redundant sequences. After removing redundant sequences from high quality isoforms, 41,293 non-redundant transcript sequences were obtained for annotation analysis (Table 1). The sequencing data is available at NCBI under the accession number of SUB6581297.

**Table 1:** Statistics of full-length transcriptome sequencing.

Statistic	Number
Clean data size	45.29Gb
Circular consensus (CCS)	475,276
Read bases of CCS	1,307,818,400
Mean read length of CCS	2,751
Full-length sequences	400,368
Consensus isoforms	76,994
Average consensus isoforms read length	2,356
High-quality isoforms	75,182
non-redundant transcripts	41,293
Transcripts with ORF	37,423
Transcripts with complete ORF	31,054
Annotated isoform number in Nr	34,751
Annotated isoform number in COG	13,648
Annotated isoform number in GO	22,854
Annotated isoform number in KEGG	21,400
Predicted lncRNA	3836
Alternative splicing events	4,307

### 3.2. Transcripts annotation

**Fig 1:**
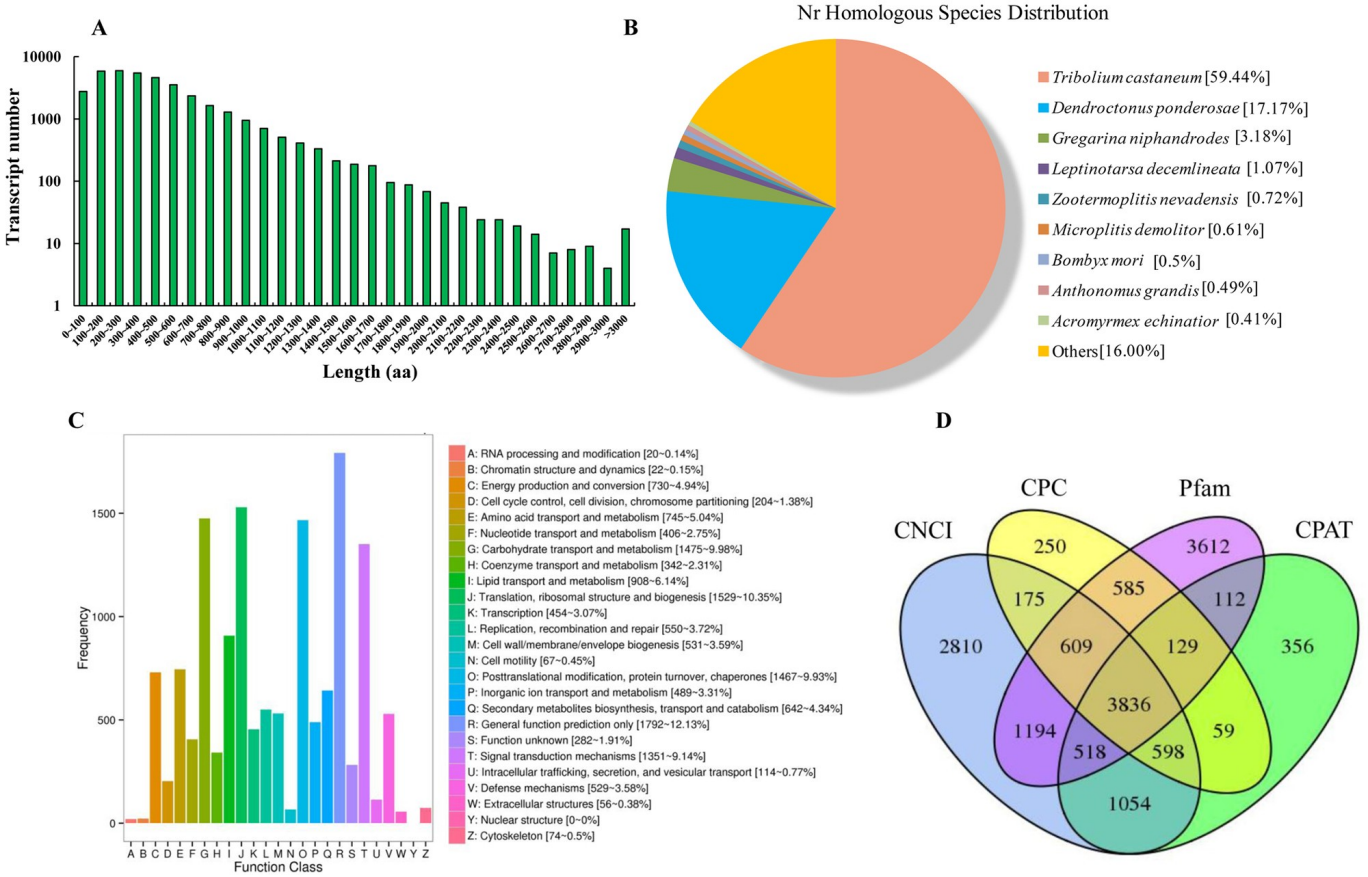
Basic information of full-length transcriptome analysis of *Phyllotreta striolata*. A. Length distribution of transcripts; B. homologous species distribution in Nr database; C. COG function classification of transcripts; D. lncRNA prediction results.

Among the non-redundant transcript sequences, there were 37,423 transcripts containing an ORF, and 31,054 of them were complete ORF (Table 1). The amino acid lengths coded by the ORF mainly ranged from 300 to 2200 aa (60.7%) (Fig 1A). Function prediction of transcripts were annotated in databases. There were 34,751, 13,648, 22,854, and 21,400 transcripts that returned valid results in Nr, COG, GO, and KEGG databases, respectively (Table 1). Blast analysis in the Nr database showed the homology distribution of full-length transcripts of *P*. *striolata*. The majority of transcripts showed high homology with *Tribolium castaneum*, which is the model species of Coleoptera, followed by *Dendroctonus ponderosae* (17.17%), while homology with other insect species were less than 5.00% (Fig 1B). It was notable that 3.18% of the transcripts were homologous with *Gregarina niphandrodes*, which is a parasite of arthropods. According to annotation results in the COG database, the transcripts were mainly enriched in function classes such as “General function” (12.13%), “Translation, ribosomal structure and biogenesis” (10.35%), “Carbohydrate transport and metabolism” (9.98%), “Posttranslational modification, protein turnover, chaperones” (9.93%), and “Signal transduction mechanisms” (9.14%) (Fig 1C). Alternative splicing (AS) analysis was applied to identify multiple splicing methods of pre-mRNA generated by gene transcription. A total of 4,307 alternative splicing events were recorded (Table 1). Details of alternative splicing events were presented in S1 File. Besides the coding transcripts, lncRNA was also predicted. The results integrated the most widely used methods of coding potential analysis, including CPC, CNCI, Pfam, and CPAT analysis. A total of 3,836 lncRNA transcripts were predicted by all four methods (Fig 1D). Target prediction of lncRNA was presented in S2 File. Specific alternative splicing events and lncRNA related to detoxification genes were further screened out.

### 3.3. Full-length transcripts of P450 genes (P450s), and related lncRNA and AS events

**Fig 2:**
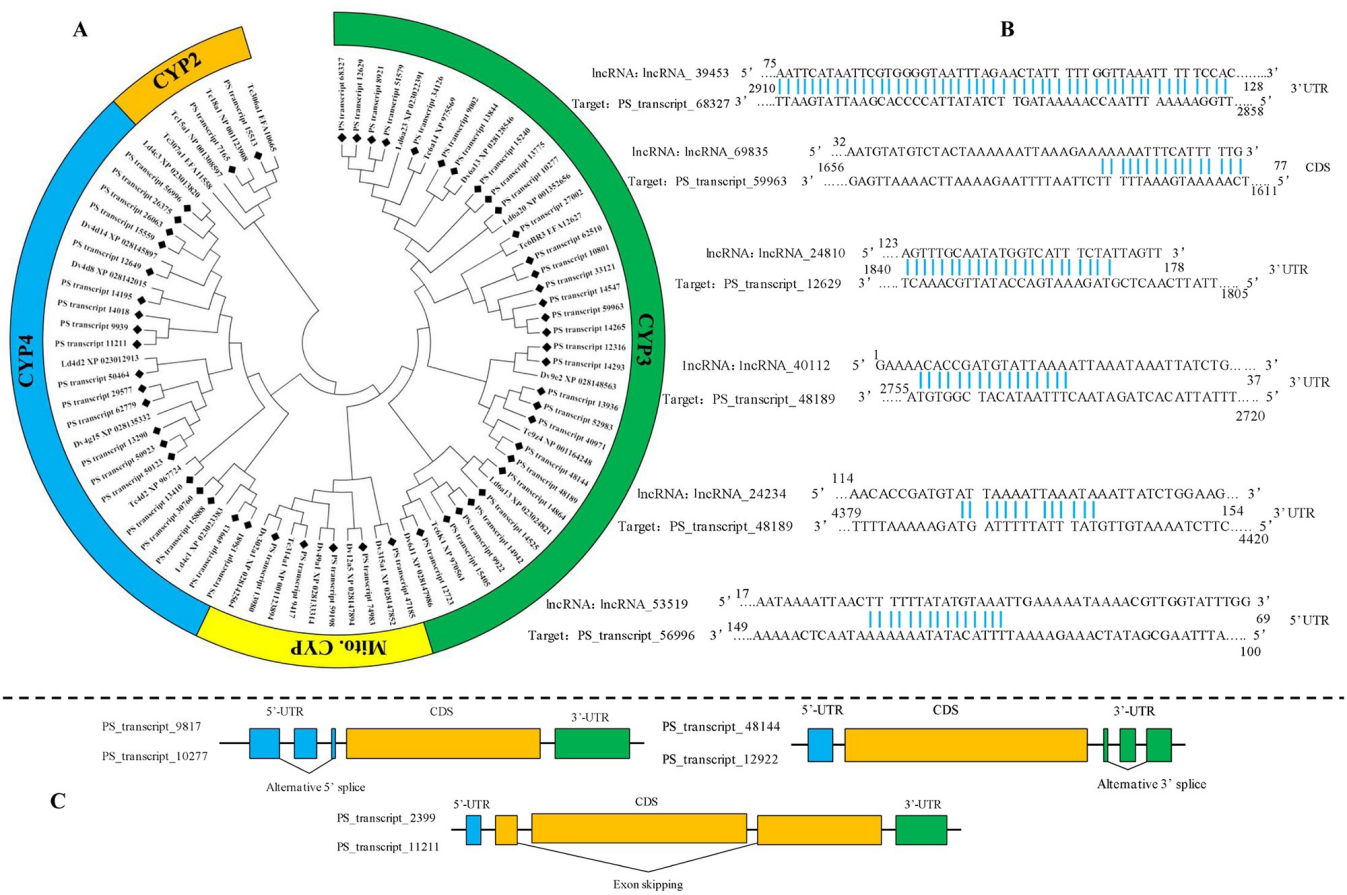
Sequence analysis, regulatory lncRNA, and splicing events of P450s. A. Phylogenetic analysis of transcripts encoding P450s; B. binding sites of lncRNA and P450s; C. splicing events of P450s.

According to function annotation results in all databases, a total of 188 transcripts with a coding region of P450 were screened out. The completeness of these transcripts was manually confirmed by comparison of amino acid sequence in the nr database. Duplicates of the same gene were also removed by cross comparison. After removal of incomplete sequences and duplicates, 57 transcripts with the full coding region of P450 were identified. The mean length of fully sequenced ORFs was 1491 bp. Phylogenetic analysis based on amino acids of other Coleoptera showed that 57 P450 coding transcripts distributed in four clades, CYP2, CYP3, CYP4, and mitochondrial, in which CYP3 was the largest clade containing 30 transcripts (Fig 2A). Most transcripts were enriched in the cyp6, cyp9, and cyp4 families. Genes in these families generally function in detoxification of exogenous toxins. Another important function of P450 in insects is biosynthesis of ecdysone. *CYP302*, *CYP306*, *CYP307*, *CYP314*, *CYP315*, *CYP18* are involved in this process and known as Halloween genes. The full transcriptome of *P*. *striolata* detected five P450 genes having high homology with the Halloween genes (*CYP302*, *CYP306*, *CYP314*, *CYP315*, *CYP18*), while one gene (*CYP307*) was missing. The sequences of Halloween genes and *PS_transcript_68327* from cyp6 family were verified by PCR. The sequences alignment showed high reliability of trascriptome data (Table 2). Although the PCR product of one Halloween gene, *CYP314*, showed a correct electrophoresis band, no signal was detected when sequencing with either universal or specific primers.

**Table 2:** Sequence validation by PCR.

Gene name/Transcriptome ID	GenBank accession numbers	Sequence identity with transcriptome data	
		Nucleotides	Amino acids
CYP18/PS_transcript_7165	MW149298	100%	100%
CYP302/PS_transcript_13980	MW149299	100%	100%
CYP306/PS_transcript_15513	MW149300	99.18%	98.76%
CYP315/PS_transcript_47185	MW149301	99.20%	99.13%
CYP6a13/PS_transcript_68327	MW149302	99.87%	99.61%
ace1/PS_transcript_444	MW149303	100%	100%
ace2/PS_transcript_75978	MW149304	100%	100%
CarE B/PS_transcript_60296	MW149305	99.56%	99.62%
ABC G1/PS_transcript_61690	MW149306	97.11%	97.36%
ABC D1/PS_transcript_5581	MW149307	99.48%	99.87%
GST sigma1/PS_transcript_20971	MW149308	99.69%	100%
GST epsilon1/PS_transcript_15201	MW149309	99.54%	98.62%
UDP1/PS_transcript_31014	MW149310	99.74%	99.80%
UDP2/PS_transcript_14115	MW149311	99.68%	99.42%

The lncRNA can function as a regulator of mRNA and the transcriptome analysis revealed six lncRNA targeting five P450 genes (Fig 2B). The *lncRNA_53519* could bind to the 5’UTR of the *PS_transcript_56996*, and *lncRNA_69835* could bind to the CDS of *PS_transcript_59963*, and the binding site of the other four lncRNA was located at 3’UTR of the P450 genes. Notablely, *lncRNA_40112* and *lncRNA_24234* were both predicted as regulators of *PS_transcript_56996*, and the binding sites are located in 3’UTR.

Two alternative splicing (AS) events were detected in the P450 transcripts of *P*. *striolata*. *PS_transcript_10277* and *PS_transcript_12922* were annotated as alternative splicing of *PS_transcript_9817* and *PS_transcript_48144*, respectively. Compared with *PS_transcript_9817*, mRNA sequence of *PS_transcript_10277* skipped a region of 171 bp in 5’UTR. *PS_transcript_12922* skipped a region of 107 bp in 3’UTR of *PS_transcript_48144* (Fig 2C).

### 3.4. Full-length transcripts of esterase genes (ESTs) and related lncRNA and AS events

**Fig 3:**
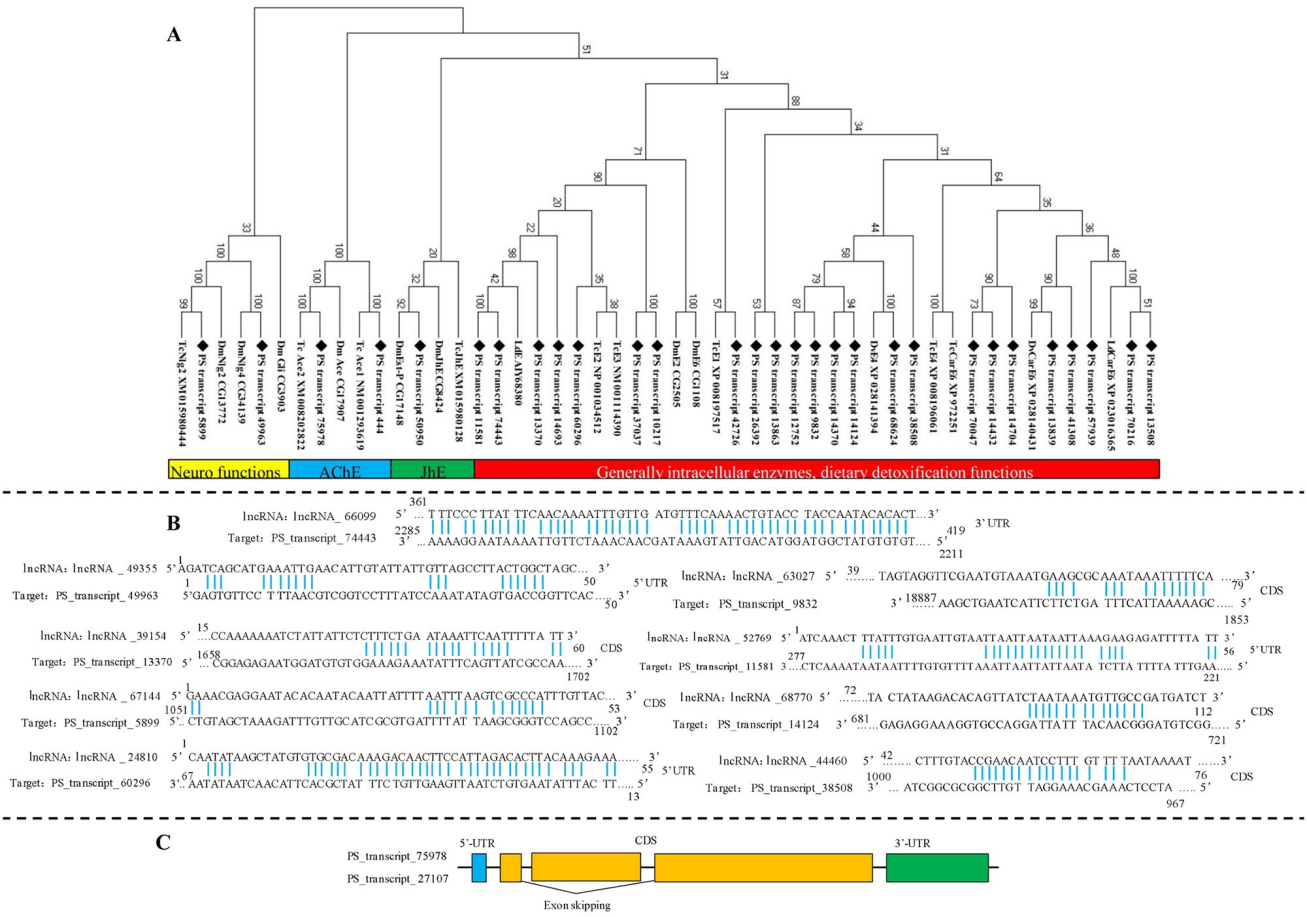
Sequence analysis, regulatory lncRNA, and splicing events of ESTs. A. Phylogenetic analysis of transcripts encoding ESTs; B. binding sites of lncRNA and ESTs; C. splicing events of ESTs.

Nighty-eight transcripts with a coding region of esterase from the transcriptome data were manually submitted to blast in the nr database. After removing incomplete sequences and duplicates, 29 identical full length transcripts encoding esterase were varified. The mean length of fully sequenced ORFs was 1747 bp. A phylogenetic tree classified the 29 transcripts into four functional groups (Fig 3A). “Generally intracellular enzymes, dietary detoxification functions” was the major group containing 24 transcripts. Eight of them clustered in the branch of detoxification enzyme, “carboxylesterase”, and the others may take part in generally intracellular and digestion processes. Two transcripts functioned as “AChE”, which is an important target of some insecticides. *PS_transcript_444* and *PS_transcript_75978* were annotated as homology genes of *ace1* and *ace2* of *T*. *castaneum*. Two transcripts showed high homology with neuroligin of *T*. *castaneum*, and *D*. *melanogaster* (“Neuro functions”). *PS_transcript_50950* in “JhE” is a crucial enzyme for insect development. The sequences of two ace genes (*PS_transcript_444*, *PS_transcript_75978*) and a carboxylesterase gene (*PS_transcript_60296*) were varfied by PCR. The sequences alignment showed high reliability of trascriptome data (Table 2).

The lncRNA analysis identified nine lncRNA targeting nine esterase genes (Fig 3B). Two neuroligin genes both had regulatory lncRNA. The *lncRNA_49355* could bind to *PS_transcript_49963* at 5’UTR, while *lncRNA_67144* targeted CDS of *PS_transcript_5899*. The targets of the other seven lncRNAs were all general esterases, and four binding sites were in CDS, two in 5’UTR, and one in 3’UTR.

Only one AS event was detected in the esterase transcripts of *P*. *striolata*. *PS_transcript_27107* was annotated as alternative splicing of *PS_transcript_75978*, and it skipped a region of 558 bp in CDS (Fig 3C).

### 3.5. Full-length transcripts of glutathione S-transferase genes (GSTs), and related lncRNA and AS events

**Fig 4:**
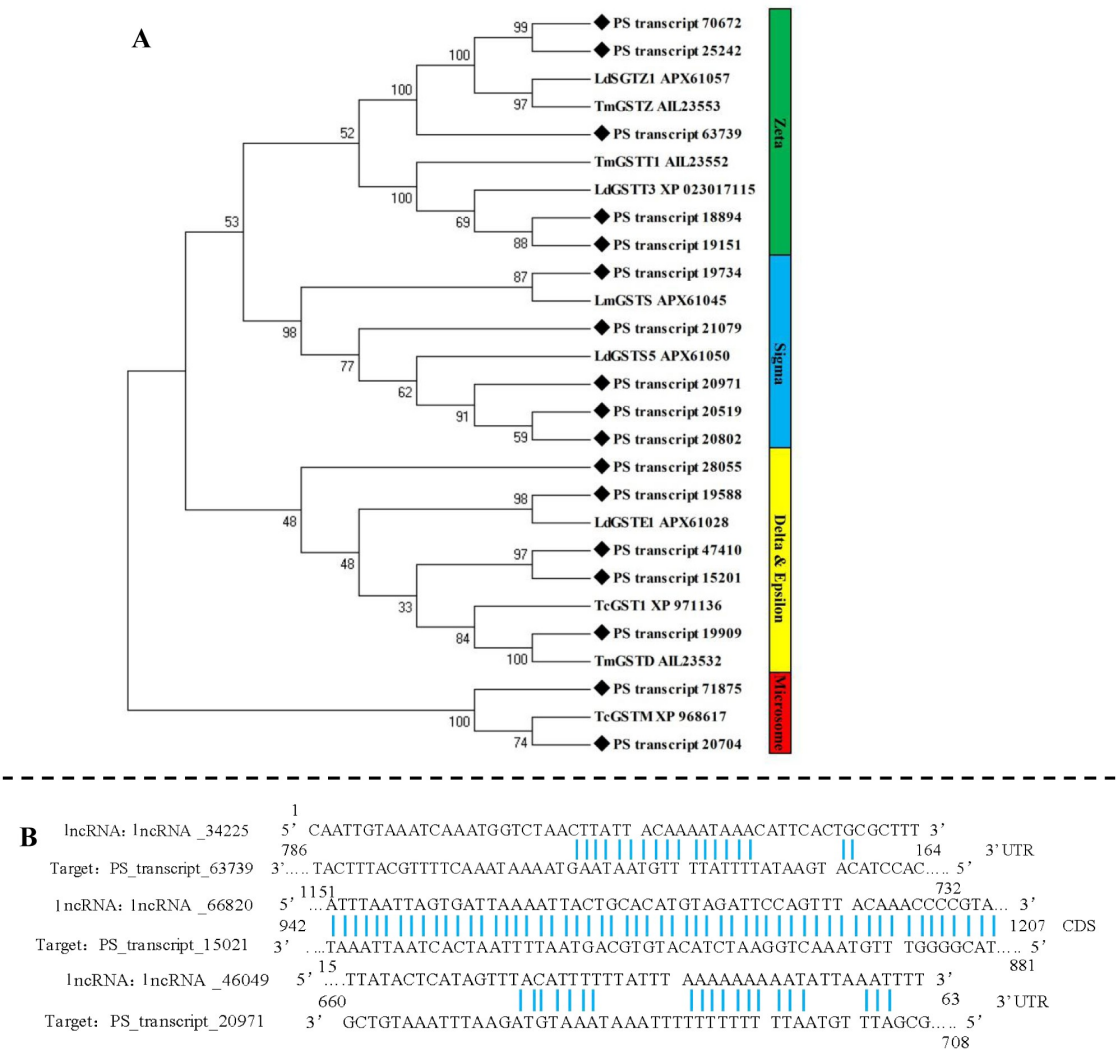
Sequence analysis, regulatory lncRNA, and splicing events of GSTs. A. Phylogenetic analysis of transcripts encoding GSTs; B. binding sites of lncRNA and GSTs; C. splicing events of GSTs.

In the transcriptome data, 35 transcripts were annotated as GSTs, after removing incomplete sequencse and duplicates, 17 full-length sequences of GSTs were confirmed. The mean length of fully sequenced ORFs was 611 bp. These 17 genes were clustered into four groups and five classes including “Delta and Epsilon” (5 transcripts), “Zeta” (5 transcripts), “Sigma” (4 transcripts), and “Mirosome” (5 transcripts) (Fig 4A). The sequences of *PS_transcript_20971* from sigma class and *PS_transcript_15201* from epsilon class were varfied by PCR. The sequences alignment showed high reliability of trascriptome data (Table 2).

Three lncRNA targeting GST genes were screened out (Fig 4B). The *lncRNA_34225* could bind to a Zeta Gst gene (*PS_transcript_63739*) at 3’UTR, and *lncRNA_46049* could bind to 3’UTR of a Delta Gst gene (*PS_transcript_20971*). The *lncRNA_66820* targeted the CDS of a Sigma GST (*PS_transcript_15201*).

No alternative splicing (AS) event was detected in the GST transcripts of *P*. *striolata*.

### 3.6. Full-length transcripts of ABC transporter genes (ABCs), and related lncRNA and AS events

**Fig 5:**
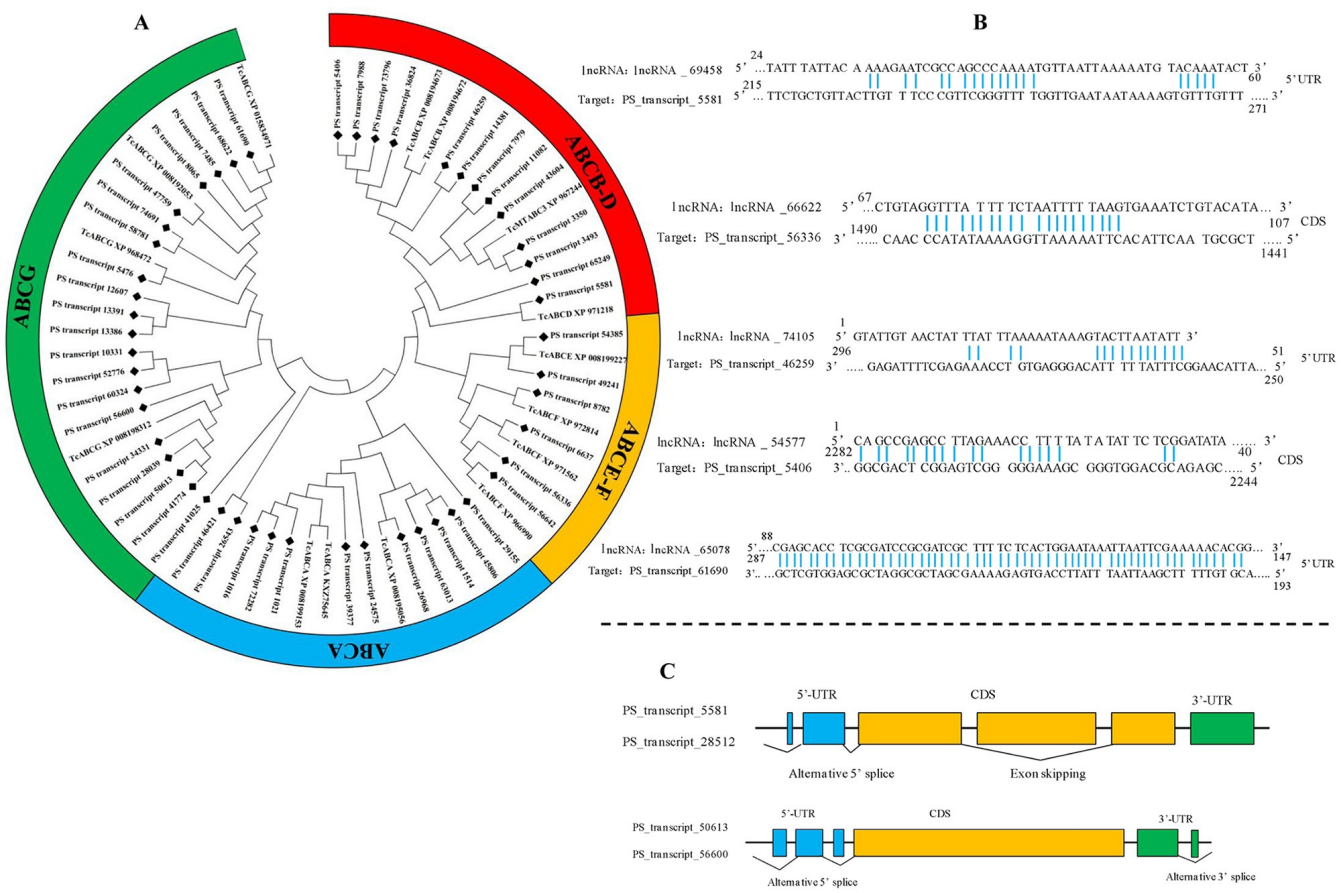
Sequence analysis, regulatory lncRNA, and splicing events of ABCs. A. Phylogenetic analysis of transcripts encoding ABCs; B. binding sites of lncRNA and ABCs; C. splicing events of ABCs.

There were 135 transcripts encoding ABC transporters in the transcriptome data. After removing incomplete sequences and duplicates, 51 full-length ABC sequences were confirmed. The mean length of fully sequenced ORFs was 2562 bp. Phylogenetic analysis showed that the ABC transcripts were distributed in seven families including “A” (12 transcripts), “B” (6 transcripts), “C” (5 transcripts), “D” (2 transcripts), “E” (2 transcripts), “F” (4 transcripts), and “G” (20 transcripts) (Fig 5A). The sequences of *PS_transcript_61690* from G family and *PS_transcript_5581* from D family were varfied by PCR. The sequences alignment showed high reliability of trascriptome data (Table 2).

Five lncRNA were identified as regulators of ABC genes (Fig 5B). The *lncRNA_69458*, *lncRNA_74105*, *lncRNA_65078* could bind to the 5’UTR of *PS_transcript_5581*, *PS_transcript_46259*, and *PS_transcript_61690*, respecitively, while *lncRNA_66622* and *lncRNA_54577* could bind to the CDS of the *PS_transcript_56336* and *PS_transcript_5406*.

Two alternative splicing (AS) events were detected (Fig 5C). “Alternative 5’ splice” and “Exon skipping” were both identifed in *PS_transcript_28512*, which was annotated as alternative splicing of *PS_transcript_5581*. Two sites of “Alternative 5’ splice” and one site of “Alternative 3’ splice” were identified in *PS_transcript_50613*, which was annotated as alternative splicing of *PS_transcript_56600*.

### 3.7. Full-length transcripts of UDP-glucuronosyltransferases genes (UGTs), and related lncRNA and AS events

**Fig 6:**
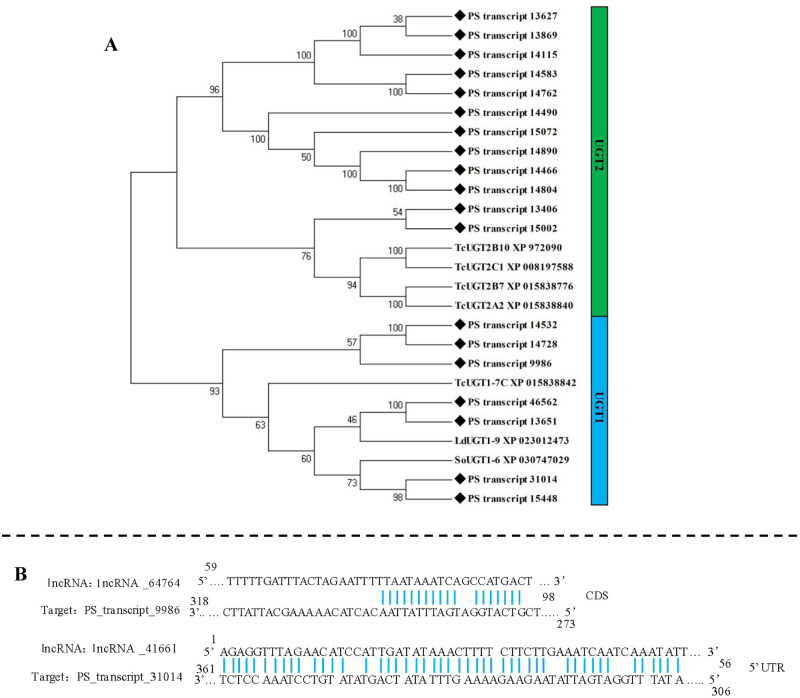
Sequence analysis, regulatory lncRNA, and splicing events of UGTs. A. Phylogenetic analysis of transcripts encoding UGTs; B. binding sites of lncRNA and UGTs; C. splicing events of UGTs.

Seventy-one transcripts were annotated as UGT genes. After removing incomplete sequences and duplicates, 19 full-length sequences of UGTs were confirmed. The mean length of fully sequenced ORFs was 1556 bp. These UGTs were clustered into two families, including “UGT1” (7 transcripts), and “UGT2” (12 transcripts) (Fig 6A). The sequences of *PS_transcript_31014* from UGT1 family and *PS_transcript_14115* from UGT2 family were varfied by PCR. The sequences alignment showed high reliability of trascriptome data (Table 2).

Two lncRNAs were identified as regulator of UGTs (Fig 6B). The *lncRNA_41661* could bind to the 5’UTR of *PS_transcript_31014*, and *lncRNA_64764* could bind to the CDS of *PS_transcript_9986*.

No alternative splicing (AS) event was detected in UGT transcripts of *P*. *striolata*.

## 4. Discussion

This full transcriptome analysis of *P*. *striolata* revealed 37,423 transcripts with ORF and 34,751 were annotated in the Nr database. Homology analysis of the transcripts showed that most of the transcripts had high homology with the sequences of *T*. *castaneum*, which is the model Coleopteran with a fully sequenced genome [25]. In this case, sequences from *T*. *castaneum* were used as an important reference to analyze the identified transcripts of *P*. *striolata*.

Multiple sequences were classified as P450s, which is a large superfamily. They function in detoxification and biosynthesis pathways [26]. The accurate number of P450s in different species varies. According to genome data, there are 204 P450s in *Culex quinquefasciatus*, while only 38 exist in *Pediculus humanus humanus* [27,28]. A total of 143 genes encoding P450s were identified in *T*. *castaneum*, with 133 being putatively functional isoforms and the other 10 being pseudogenes. Phylogenetic analysis showed that CYP3 and CYP4 are two major clans and cyp4, cyp6, cyp9 are three important families within these two clans [29]. A total of 188 transcripts encoding P450s were screened out from the full transcriptome of *P*. *striolata*. After removing partial and duplicate sequences, we only found 57 fully sequenced transcripts. These transcripts were mainly distributed in the CYP3 and CYP4 clans and cyp4, cyp6, cyp9 families as well. In insects, P450 genes in the cyp6 family are widely involved in pesticide detoxification [30–32]. This is also the largest family of *P*. *striolata* identified in this study including 23 unique transcripts. It will be a useful resource for P450s functioning in detoxification with a possible direct relationship with the insecticide resistance mechanism of *P*. *striolata*. Besides detoxification, a group of special P450s in arthropods, called Halloween genes, are involved in ecdystroid biosynthesis [33]. Most full sequences of the Halloween genes were identified here, but *CYP307* was missing as well as any partial sequences. In some arthropod species Halloween genes have not been found [34]. However, Coleoptera generally have a complete sets of Halloween genes including *CYP307*. Thus, failure to find *CYP307* in *P*. *striolata* may be due to blind areas in the transcriptome sequencing [29,35].

Genes with functions as generally intracellular, dietary, and detoxification enzymes form the largest group of esterases in *P*. *striolata*. As a kind of particular esterase, acetylcholinesterase (AChE) of insects is an important target of many insecticides, and most insect species have two AChE genes. *T*. *castaneum* and *P*. *striolata* were not exceptions to this rule. The first AChE gene (*ace*) was sequenced from *D*. *melanogaster*, and it is the only sequence encoding AChE in *D*. *melanogaster*. *Ace1* from other insects is considered as the paralogous gene of *ace*, and *ace2* is considered as orthologous to *ace* [36]. Functional analysis of these two genes indicates that *ace1* is the target site of anticholinesterase insecticides, while the importance of *ace2* is comparatively less [37]. This study found both fully sequenced *ace1* and *ace2* genes in *P*. *striolata* as well as a alternative splicing sequence with an exon skipping in the CDS of *ace2*.

As an important phase II detoxification enzyme, GSTs are mainly involved in the detoxification of pesticides. In the *T*. *castaneum* genome, a total of 41 GST genes were anotated. Delta and Epsilon were the two biggest classes of GSTs, and their function is highly correlated to insecticide resistance [38]. However, according to the transcriptome of *P*. *striolata*, 35 transcripts were identified as GSTs, including 17 full-length sequences of GSTs.

Compared to P450s, CarEs, and GSTs, which are important detoxification enzymes, ABCs and UGTs are relatively newly identified groups that also have a role in insecticide resistance [39,40]. In the transcriptome analysis of *P*. *striolata*, 135 transcripts encoding the ABC transporter were identified, and 51 were full-length sequences, distributed in 7 families. A total of 73 ABCs have been annotated in the genome of *T*. *castaneum*. ABCC is the largest family and it is mainly associated with resistance. ABCs are involved in many important physiological processes. RNAi of ABCs in *T*. *castaneum* can cause aberrant phenotypes such as wing, molting and developmental defects, white eyes, and death before molting [41]. This suggests that ABCs are potential RNAi targets for developing novel control methods for *P*. *striolata*.

As GSTs, UGTs are phase II detoxification enzymes widely distributed in various insect species. UGTs have been divided into two distinct families, UGT1 and UGT2, based on sequence identities [42]. UGTs from the UGT2 family are mainly involved in detoxification of xenobiotics. In insects, over-expression of UGT2 genes is usually related to insecticide exposure or resistance [43,44]. A total of 43 UGTs have been classified in the genome of *T*. *castaneum* [45]. In this study, 71 transcripts were annotated as UGTs, and 19 sequences had a complete coding region. Most of these belong to the UGT2 family.

Although up-regulation of expression and specific point mutation were most studied mechanisms for detoxification resistance of insects [46], the alternative splicing of detoxification genes has attracted much interest with development of sequencing technology [47]. Besides providing information on the complete sequences of functional genes, another advantage of TGS technology is prediction of alternative splicing events. A total of six alternative splicing events were observed in classified genes, three in P450s, two in ABCs, and one in ESTs. There are three kinds of alternative splicing events according to their position. These include alternative 5’-UTR or 3’-UTR splice, and direct skipping in the CDS. Splicing in the 5’-UTR or 3’UTR may affect the post transcriptional regulation of a specific gene and result in a higher or lower translation to functional protein. Skipping in the CDS can lead to a direct function change. The three alternative splicing events of P450s were identified in 5’-UTR, 3’UTR, and CDS, respectively, which might change the translation of mRNA or directly affect the enzyme activity. In the *Nilaparvata lugens*, a novel alternative transcript of *CYP6ER1* was found important for imidacloprid resistantance [48]. In *Drosophila*, an alternative splicing event in ABC gene *MDR49* was associated with DDT resistance [49]. In this study, two alternative splicing events were detected in ABCs of *P*. *Striolata*, which might also contribute to inseciticide resistance. Only one alternative splicing event was found in ESTs, and interestingly, it was happened in AchE gene, which was not a detoxificaiton enzyme, but a target of organophosphorus insecticide. The target change could lead to high resistance level, which were commonly occured in insects [50]. As splicing is a major mechanism for the enhancement of transcriptome and proteome diversity [51], these data would provide a broad view to understand detoxification mechanism of *P*. *Striolata*.

The lncRNA is a regulator of gene expression. It is involved in nearly every level of the gene expression program. The lncRNA can participate in posttranscriptional gene regulation through controlling processes like protein synthesis, RNA maturation, and transport as well as in transcriptional gene silencing through regulation of the chromatin structure [52]. The full transcriptome analysis also predicts lncRNAs based on their interaction with sequences of transcripts, and these information could be used in analysis of detoxification mechanisms of insects. In the studies of *Plutella xylostella*, lncRNAs associated with P450s, ESTs, UGTs, ABCs and insecticide targets were sequenced and annotated. The function of these lncRNAs was thought to contribute to chlorantraniliprole and BT resistance [53,54]. In this case, the predicted lncRNAs in *P*. *Striolata* will provide insights into the regulation of detoxification genes.

## 5. Conclusions

Our data provide abundant gene resources with complete CDS for function analysis, and its accuracy is confirmed by PCR validation. It suggests that full-length transcriptome sequencing is an efficient way to promote molecular study of organisms without genome.

## Supporting information

S1 Table Primer information. (DOC)

S1 File Predicted alternative splicing events. (XLS)

S2 File Predicted targets of lncRNA. (XLS)

S3 File Sequence information of full-CDS of P450s. (XLS)

S4 File Sequence information of full-CDS of Ests. (XLS)

S5 File Sequence information of full-CDS of GSTs. (XLS)

S6 File Sequence information of full-CDS of ABCs. (XLS)

S7 File Sequence information of full-CDS of UGTs. (XLS)

S8 File Sequence information of lncRNAs. (XLS)
