# Supplementary material for: Full length sequencing reveals novel transcripts of detoxification genes along with related alternative splicing events and lncRNAs in Phyllotreta striolata
Source: PLoS One. 2021 Mar 24;16(3):e0248749. doi: 10.1371/journal.pone.0248749 (PMC7990184; doi:10.1371/journal.pone.0248749)
Supplement: S1 Table — (DOC) [file pone.0248749.s001.doc]

*Supplementary Table 1 Primer information*

| Gene name | Forward primer | Reverse primer |
| --- | --- | --- |
| PS_transcript_7165 | ATGTTCGTGTACAGTACGTT | CTAATGGCTGCCAGCCTGTC |
| PS_transcript_13980 | ATGGACCTTTTTAAACGTAA | CTACGAGTCCCTTTTCATAAA |
| PS_transcript_15513 | ATGATGTTGGCAGTACTGGT | CTACAGTTCTTTTAGAAGGAA |
| PS_transcript_47185 | ATGTACAGGCTCGCCTGCAG | CTAAAGTTTATTGAAAACCAGC |
| PS_transcript_68327 | ATGGAGTTAATTTCATTAAT | TTACTTTTCCAAACGTTCC |
| PS_transcript_444 | ATGAAGAAGAAAACGATGAAGCT | TTATTGGAACGGTTCCCACATC |
| PS_transcript_75978 | ATGGTGCGATTAGTCGTGTTG | TTACGGCGCCCTCGACGATT |
| PS_transcript_60296 | ATGCGGGAACCGTTGGTGAA | CTACAATTTGGACGTGGCAA |
| PS_transcript_61690 | ATGGCGATGGAGATGCAATC | TCACCGAGCCAACCCCAGAT |
| PS_transcript_5581 | ATGCCGGCGGTGATTTCGA | CTATTCGTCCTCTCCGAGC |
| PS_transcript_20971 | ATGCCGTCCAATTTCAAGTTA | TTACAAATCGCTTATCAAAGA |
| PS_transcript_15201 | ATGGCTCCGTCTTTGTACTC | CTAATTAGCTAATTTGCTC |
| PS_transcript_31014 | ATGAATTTTGTTCTTCTATTAC | TCAATCGCTCTTATTCTTCTT |
| PS_transcript_14115 | ATGTACAGTTCGAAAACAATG | TCAATTCTTCTTTAATTTACG |
